# Robust Synchronization of Ambient Vibration Time Histories Based on Phase Angle Compensations and Kernel Density Function

**DOI:** 10.3390/s22228835

**Published:** 2022-11-15

**Authors:** Salman Saeed, Luc Chouinard, Sikandar Sajid

**Affiliations:** 1 National Institute of Urban Infrastructure Planning, University of Engineering & Technology, Peshawar 25120, Pakistan; 2 Department of Civil Engineering, McGill University, Montreal, QC H3A 0C3, Canada; 3 Department of Civil Engineering, University of Engineering & Technology, Peshawar 25120, Pakistan

**Keywords:** operational modal analysis, ambient vibration tests, system identification, singular value decomposition, kernel density function, data synchronization

## Abstract

The output-only modal analysis is ubiquitously used for structural health monitoring of civil engineering systems. The measurements for such applications require the use of multiple data acquisition systems (DAS) to avoid complicated meshes of cables in high-rise buildings, avoid traffic constriction on a bridge during measurements, or to avoid having limited channels in a single DAS. Nevertheless, such requirements introduce time synchronization problems which potentially lead to erroneous structural dynamic characterization and hence misleading or inconclusive structural health monitoring results. This research aims at proposing a system-identification-based time synchronization algorithm for output-only modal analysis using multiple DAS. A new procedure based on the compensation of the phase angle shifts is proposed to identify and address the time synchronization issue in ambient vibration data measured through multiple DAS. To increase the robustness of the proposed algorithm to the inherent inconsistencies in these datasets, the kernel density function is applied to rank multiple time-shift estimates that are sometimes detected by the algorithm when inaccuracies exist in the data arising from low signal-to-noise ratio and/or presence of colored noise in the ambient excitations. First, the synchronized ambient vibration dataset of a full-scale bridge is artificially de-synchronized and used to present a proof of concept for the proposed algorithm. Next, the algorithm is applied to ambient vibration data of a 30-story, reinforced concrete building, where the synchronization of the data could not be achieved using two DAS despite best efforts. The application of the proposed time synchronization algorithm is shown to both detect and correct the time synchronization discrepancies in the output-only modal analysis.

## 1. Introduction

Ambient-vibration (AV)-based operational modal analysis for the dynamic characterization and structural health monitoring of structures is ubiquitous in civil engineering [1,2]. One of the major challenges associated with AV testing in the output-only modal analysis is to obtain measurements from all sensing nodes simultaneously or time synchronized [3,4]. Time synchronization discrepancies can lead to erroneous and misleading results [5,6]. The effects of time synchronization discrepancies on modal analysis are detailed by many, for example Krishnamurthy et al. [5], Sim et al. [7], and Li et al. [8].

For simultaneous measurements of vibrations at different sensing nodes and hence avoiding time synchronization issues, it is required that all sensors must be connected to a single DAS. However, this can lead to complicated meshes of (sometimes very long) cables, which is intrusive and can potentially disrupt normal building activities because of the cables running through several floors as well as being time consuming to implement [9]. Furthermore, the use of long cables can result in a low signal-to-noise ratio, which is already an issue with AV tests. Alternatively, multiple DAS can be employed to eliminate the use of long cables. This reduces the cost and increases the efficiency of the test significantly, making it possible to plan health monitoring measurements with minimal disturbance to the occupants and operations in the building. Multiple DAS may also be employed in situations where the channels in a single data acquisition are not sufficient for the planned measurements [10]. However, the data thus obtained can have synchronization discrepancies since the data acquisitions need to be triggered at the exact same time, i.e., within the sampling time period (close to tens of milliseconds in most applications) of the data acquisition [11,12,13].

The challenges associated with the time synchronization issues in wireless sensor networks are reported in the current state of the art [14,15,16,17]. Several approaches have been proposed to address these issues in measurements, mainly for wireless sensor networks [18,19,20]. For example, synchronization with GPS time markers [3] was successfully employed with a wireless sensor network for monitoring a suspension bridge [21]. Similarly, the number of existing clock synchronization protocols for wireless sensor networks are reviewed in Rhee et al. [22]. Another successful attempt to achieve time synchronization with wireless sensor networks deployed in structures with limited satellite connectivity was implemented by Koo et al. [23] using GPS time stamps and Arduino^®^. The Arduino^®^ acts as an internal timer and the proposed method works independently on each node without exchanging time-sync packets among nodes [23]. Time synchronization issues in wireless sensor networks can also arise from ambient temperature variations, which can occur during health monitoring campaigns. Li et al. [8] propose a nonlinear clock drift compensation to achieve time synchronization to compensate for temperature effects. Dragos et al. [24] present a synchronization procedure based on minimizing differences between resonant frequencies obtained from the acceleration response spectra and those from a numerical model of the same system. Yang et al. [25] address synchronization discrepancies in multiple channel wired sensing networks by first minimizing phase slopes of the multiple channel response histories followed by maximizing the linear dependency of the modal components. Aruajo et al. [9] propose a wireless synchronization module based on the IEEE 802.15.4 protocol standard to limit the time synchronization error to less than 120 µs as they report that structural identification accuracy is influenced beyond this number especially in higher modes.

The current state of the art also reports several studies presenting the time synchronization techniques adopted for the multiple DAS. Maes et al. [26] report achieving offline time synchronization of multiple DAS by maximizing the correlation between accelerations obtained at the sensing nodes. Amador et al. [27] addressed the time synchronization issues in the multiple DAS by calculating the transfer functions that relates the measurement signals obtained. Maes et al. [28] report addressing the synchronization issue in multiple DAS using system identification characteristics for an input–output structural health monitoring application.

This research presents a comprehensive study on the effects of time synchronization discrepancies in the output-only modal analysis and proposes a new correction procedure that enhances the robustness of structural identification techniques using multiple DAS as required by a variety of reasons explained above. First, the basis and procedure to implement the proposed algorithm are described, followed by its validation with artificially de-synchronizing a synchronized dataset obtained on a bridge, and finally with an application to the ambient vibration data of a 30-storey, reinforced concrete (RC) building, where despite best efforts, the data synchronization between two DAS could not be guaranteed.

## 2. Proposed Algorithm

**Figure 1:**
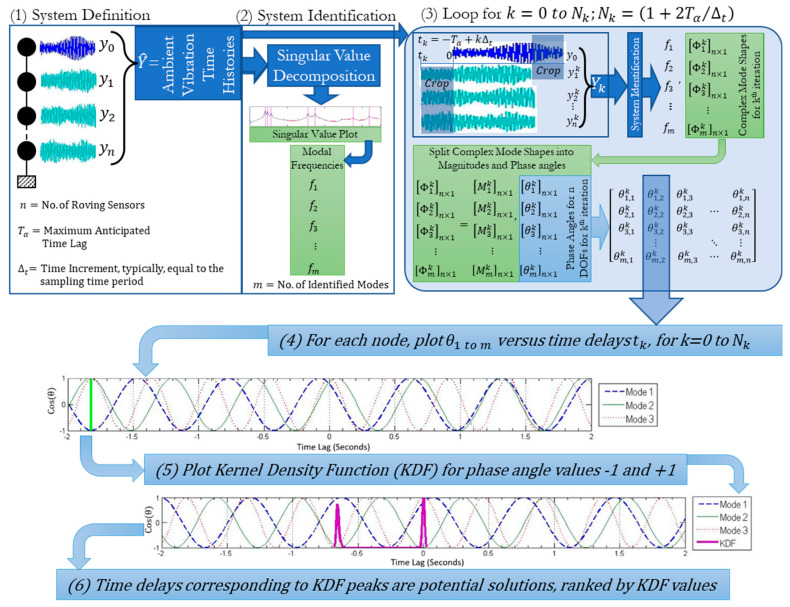
Flowchart of the proposed synchronization algorithm.

The underlying concept of the time synchronization algorithm is that phase angles at all nodes in mode shapes should be equal to either 0 or 180 degrees for the first few natural frequencies of a structure (the first three in this case, these are monotonic functions). This assertion is supported by Figure 6, where the cosines of mode shape phase angles for first and second modes obtained from a synchronized dataset of an AVT of a bridge are shown to be either −1 or +1, corresponding to phase angles of 0 or 180 degrees. The flowchart of Figure 1 illustrates the steps in the proposed algorithm. The phase angle correction at each node can be determined by shifting the time series from the reference node by small increments (typically equal to the sampling period used in data acquisition) relative to the time series from a roving node in each setup for a range of possible time lags (e.g., −2 to +2 s) (step 1). For each time lag, the shifted time series are used to assemble the system matrix (step 2). The singular value decomposition of the system matrix provides mode shapes at each node for the given time lag, while the phase angles of the complex mode shapes are extracted (step 3). The phase angles are then plotted as a function of the range of time lags for all setups (step 4). These plots are referred to as phase angle vs. time lag, or PATL, plots in this text.

The first PATL in step 4 of the flowchart in Figure 1, illustrates a test case where synchronized data was deliberately de-synchronized by 1.82 s, and the phase angles of the first three mode shapes are plotted against the time lags. The green vertical line in the PATL shows the point where the phase angles of all three mode shapes are either 0° or 180°, depicting the solution, i.e., the time lag that the data was de-synchronized by.

In some cases, due to the in-accuracies arising from various sources, such as colored excitations and low signal-to-noise ratios or due to inherent structural properties (such as damping), the peaks and troughs of the PATL of all nodes do not intersect exactly in the entire range of possible time lags. To address this issue, the kernel density function (KDF) is used to identify the most likely time lag (step 5). In the second PATL of the figure, each of the three initial modes (*j* = 1:3), the time lags (*i* = 1:*n_j_*) corresponding to 0° or 180° phase angles are first identified $\left\{\Delta t_{ij}\right\}$. The likelihood for the potential time lag is obtained as:

(1) $$\hat{f}\left(\Delta t\right)=\frac{1}{h\sum_{j=1}^{3}n_{j}}\sum_{j=1}^{3}\sum_{i=1}^{n_{j}}K\left(\frac{\Delta t-\Delta t_{ij}}{h}\right)$$

where K() is a Gaussian function with zero mean and optimized bandwidth h [29]. The KDF likelihood is plotted over the same PATL, while the possible time lag(s) is identified as peak(s) in the KDF, and if multiple peaks exist, then they are ranked in order of decreasing likelihood (step 6). In the second PATL plot in the figure, potential time lags are found as 0 and −0.65 s. The correct time lag between the two candidate lags are selected iteratively based on minimization of the deformation energy in the identified mode shapes. In most cases, there is only a single peak in the KDF plot; however, when multiple peaks exist, then the correct time lag is selected by iterating through all potential time lags in the order of decreasing likelihood and visually assessing the mode shapes resulting from each potential time lag. To process the AVT data, a MATLAB-based graphical user interface program called “SysID” was developed for system identification using frequency domain decomposition (FDD) [11] while incorporating the proposed synchronization algorithm. The complete code of this program is available in the appendix of [13]. All the figures in this paper are outputs of the SysID software.

## 3. Proof of Concept

To evaluate the performance and accuracy of the proposed algorithm, a synchronized dataset was obtained and was then de-synchronized by random time lags. The proposed algorithm is then applied to this de-synchronized data to estimate the time lags based on the phase angles of mode shapes. The performance of the algorithm can be judged by the ability to recover the synchronized dataset and by the closeness of the estimated and actual time lags.

### 3.1. Description of the Bridge and Data Collection

**Figure 2:**
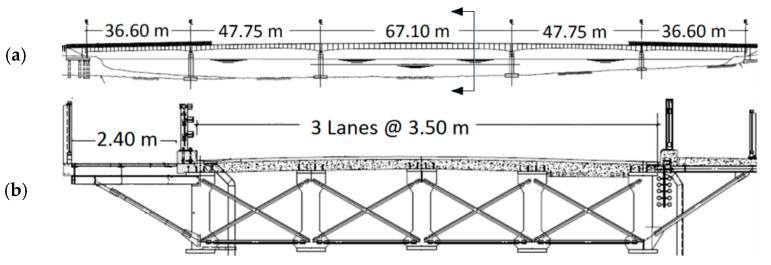
(**a**) Longitudinal view of the Jacque Bizzard bridge. (**b**) Cross-sectional view of the bridge.

Jacque Bizzard Bridge in Montreal, Quebec, Canada, was constructed in 1966 and consists of five steel girders of variable depth and length with a cast-in-place concrete deck for a sidewalk. The bridge consists of five spans; the mid span is 67.10 m, flanked by spans of 47.75 m and 36.60 m on either side (Figure 2a). The deck is 12.50 m wide with three lanes and a walkway on the downstream side. A 2.40 m wide steel bike path was added later to the upstream side of the bridge (Figure 2b).

The sensors used for this test were TROMINO^®^ velocity meters. These sensors have built-in DAS with radio transceivers for synchronization. During the tests, the TROMINOs communicate with each other to keep the internal clocks synchronized within one millisecond. A total of six sensors were used for the test. The weather conditions on the day of the test were sunny with normal wind. The traffic on the upstream side was minimal, while the traffic on the downstream side was moderate. A total of 46 equally spaced nodes were identified on the bridge, 23 on each side. The two nodes in the middle of the bridge on each side were selected as reference nodes. Four roving sensors were moved across the bridge in ten setups, five on each side of the bridge. During each setup two sets of data were recorded, each for approximately six minutes. The length of measurement selected were based on similar studies [30,31,32,33]. One of the two datasets for each node with the least levels of high excitations due to traffic, was selected for further analysis. The sampling frequency was set to 128 Hz and radio transceivers were used to trigger the start of recording simultaneously in all sensors, and to keep the sampling synchronized within one millisecond. The data thus obtained was synchronized. A separate dataset is generated by introducing artificial de-synchronization in the original dataset by a random time between +/−2 s, assuming that in absence of radio synchronization, the users would have started the sensors on visual or audio queues with accuracy within +/−2 s. The two datasets, synchronized and desynchronized, were then processed using the frequency domain decomposition (FDD) [11].

### 3.2. Development and Application of the Proposed Time Synchronization Algorithm

**Figure 3:**
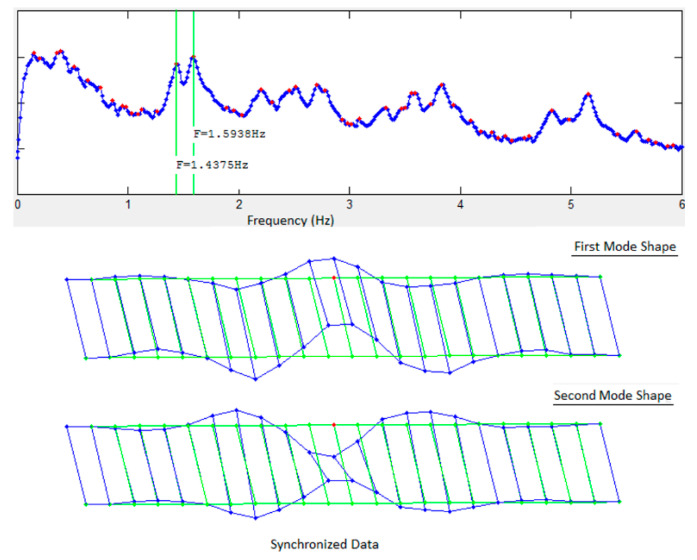
Singular value plots and first two mode shapes for synchronized data.

**Figure 4:**
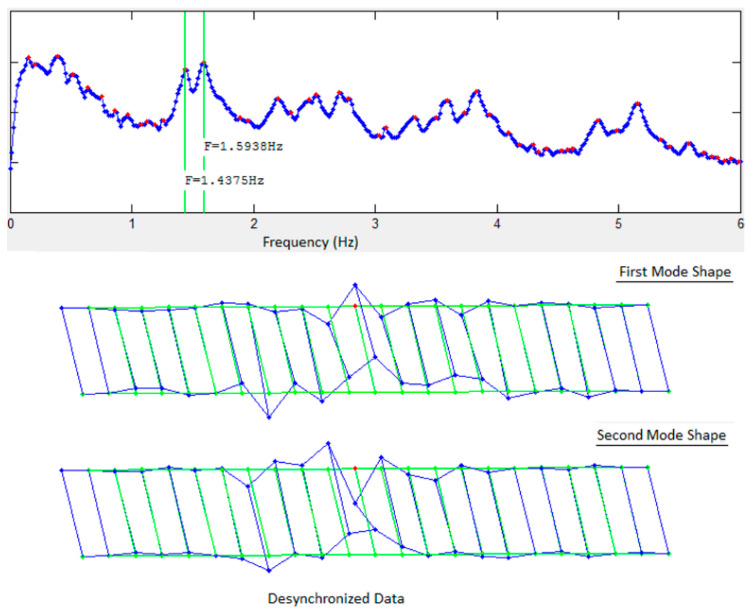
Singular value plots and first two mode shapes for de-synchronized data.

The FDD technique was used for system identification using the two datasets to generate the singular value plots (SVP) and the mode shapes. Figure 3 and Figure 4 show the first singular value plots and first two mode shapes from synchronized and de-synchronized datasets, respectively. The blue line in the SVP are the singular values, while the red dots show the local peaks, while the identified system frequencies are indicated by green vertical lines. In the mode shapes, the green lines show the undeformed model, and the blue lines show the deformed model. It can be readily noticed from the two figures that frequency content of the data is not affected by de-synchronization as the singular value plots for both datasets are identical and that natural frequencies can still be estimated correctly from the de-synchronized dataset. However, there is a significant difference in the mode shapes obtained from the two datasets. This difference in mode shape exists due to phase angle shifts imposed by time lags or de-synchronization of the data.

**Figure 5:**
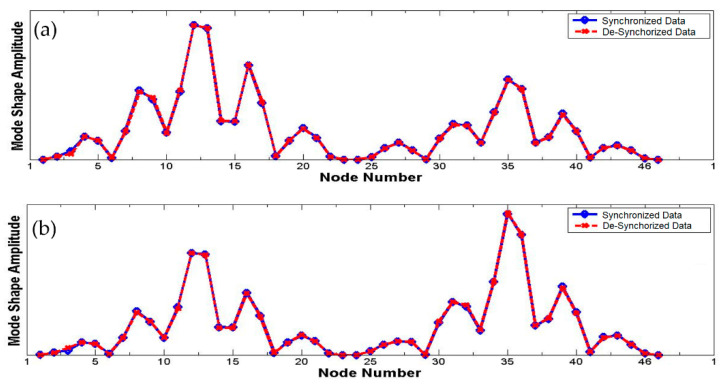
Mode shape amplitudes at each node—first mode (**a**) and second mode (**b**).

**Figure 6:**
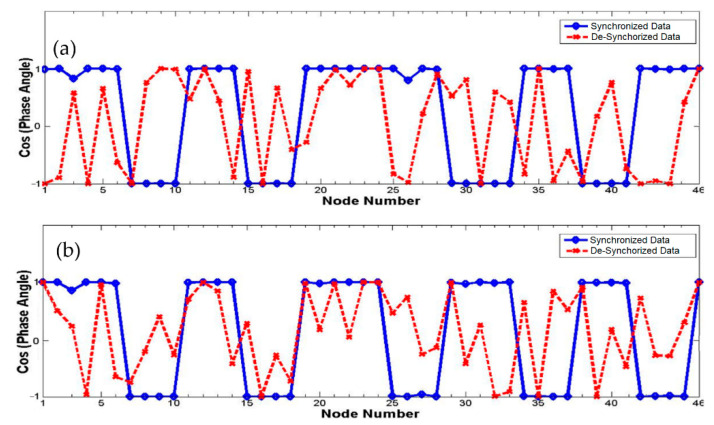
Cosine of phase angles as a function of nodes—first mode (**a**) and second mode (**b**).

Examining the mode shapes by comparing their amplitudes and phase angles for the two datasets illustrates these observations. Figure 5 compares mode shape amplitudes for the first and the second modes, while Figure 6 compares the cosines of phase angles of the mode shape for the first and the second modes. The amplitudes for both synchronized and de-synchronized datasets match exactly for first and second modes, but the phase angles in case of synchronized dataset are always close to 0 or 180 degrees, while those for de-synchronized dataset are random. The phase angles can be easily related to the time lag introduced to de-synchronize the dataset, and an estimate of this time lag can be computed and corrected for to obtain a synchronized dataset.

**Figure 7:**
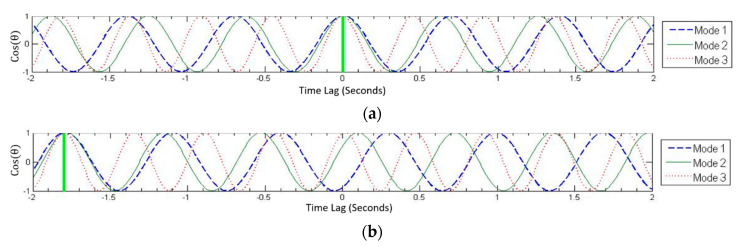
Phase angle as a function of time lag (PATL) plots for node #2 for first three modes. Synchronized dataset (**a**) and de-synchronized dataset (**b**).

Varying the time lag at each node between −2 and +2 s and determining the phase angle at each node produces a sinusoidal curve for phase angle as a function of time lag (PATL) for all modes. The crests and troughs in these plots represent possible solutions (time lags with phase angles equal to 0 or 180 degrees). The first plot in Figure 7 shows the cosine of the phase angle as a function of time lag for the first three modes of node #2. The correct time lag for the synchronized data should be 0 s since the data do not have any time lag; hence, the PATLs of all three modes can be seen coinciding at 0 s, indicated by the thick vertical line. The second plot in Figure 7 shows the PATLs for the same modes and node for de-synchronized dataset. The correct time lag can be readily identified to be −1.80 s as indicated by the thick green vertical line. Similarly, the time lags for all the nodes can be found, which when applied to data from each setup essentially synchronizes the desynchronized dataset.

**Figure 8:**
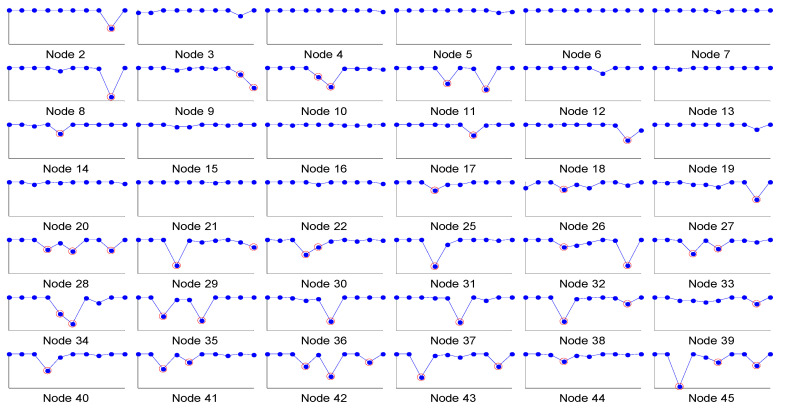
Phase angles of each node in the bridge. *x*-axis is the mode number from 1 to 10 and *y*-axis is the absolute value of cosines of phase angles ranging from 0 to 1.0. The points in red circles are the “outliers”, that is, values of less than 0.80.

**Figure 9:**
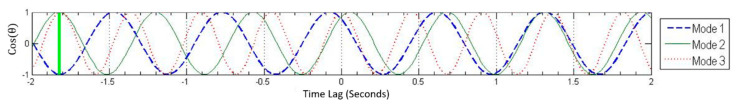
PATL plot for synchronized dataset for node #3. Notice the apparent lag in first and second modes result in the wrong solution. The correct solution should be at 0 s because data is synchronized.

Generally, the AV test datasets contain inconsistencies mainly due to low signal to noise ratio, environmental variations which can influence the internal clocks of the DAS, and variability in the loading conditions and/or operating conditions (for example, presence of heavy traffic or other factors). These inconsistencies can potentially introduce phase lags in the results even for the synchronized datasets. Such an apparent phase lag can be observed in the phase angles of nodes #3 and #26 for the synchronized dataset of the bridge as they are not exactly 0 or 180 degrees, as shown in Figure 6. This inconsistency in the data can be observed to occur at several nodes for all modes, as shown in Figure 8. The *y*-axis in Figure 8 is the absolute value of the cosine of phase angle ranging from 0 to 1.0, and the *x*-axis is the mode number starting from the first mode to tenth mode. The points with value less than 0.80 are considered outliers that result due to random inconsistencies in the data and are highlighted with red circles. It can be observed that nodes 28 and onwards have a larger proportion of outliers. These nodes were located at the downstream side of the bridge, and the bridge had a higher density of traffic during rush hours, at the time of measurements at these nodes, which may have been the cause of these inconsistencies. It can also be observed that inconsistencies may occur in any mode. Figure 9 shows the PATL plots for the first three modes of node #3 for the synchronized dataset. The inconsistencies in first and second modes of node #3 influence the PATL plots by introducing a random lag even for synchronized data.

The elimination of erroneous modes due to small drifts in PATL plots before applying the proposed algorithm can potentially address this problem. Since it is not possible to distinguish between a shift in the phase angle due to noise or lack of time synchronization when looking at a single node, it is difficult to identify these erroneous modes for the de-synchronized dataset.

To make robust estimates of the small drifts in PATL plots in certain modes, a set of potential solutions is created by considering all possible combinations of modes. Most of these combinations should produce good solutions and result in the correct time delay estimate. In theory, bad solutions are associated with noise and identify different lags that are randomly distributed.

A Kernel density function (KDF) [34] is used to obtain the distribution of available solutions. In this validation test, the distribution obtained for most of the nodes resulted in a single peak, while those tainted by noise resulted in more than one peak. The solutions obtained from the KDF of each node are then ranked according to their likelihood. Nodes with a single peak have a unique solution while those with multiple solutions are ranked according to the likelihood. To correctly identify the right solution among multiple choices, an objective criterion is the relative minimum deformation energy.

**Figure 10:**
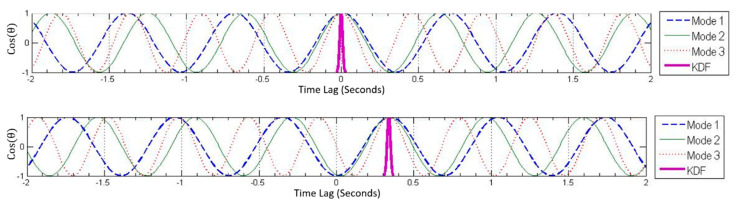
PATL superposition with KDF for node #4 in synchronized (**above**) and unsynchronized (**below**) datasets. Due to low levels of noise, the resulting KDF has only one peak at the correct solution for both datasets.

**Figure 11:**
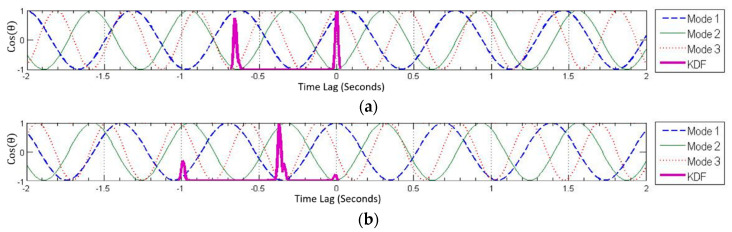
PATL superposition and KDF of node #26 (**a**) and node #45 (**b**) in synchronized data.

For most nodes in the validation test the KDF exhibits a single peak, always near the correct solution. Figure 10 shows the superimposed PATLs for node #4 in synchronized and unsynchronized datasets. Note a single, clear peak of the KDF in both plots, at 0 s for synchronized dataset and at 0.33 s for the de-synchronized dataset. Nodes with noise have multiple peaks in the KDF of solutions; however, the highest peak will almost always correspond to the correct solution. For example, node #26 in the first plot of Figure 11 shows two peaks in KDF due to bad PATLs in mode 1 for the synchronized dataset, but the higher peak is at 0 s, which is the correct solution.

In extreme cases, it is possible that some nodes may have several corrupted PATLs. In this case, the KDF may have multiple peaks and the highest peak may not correspond to the correct solution; rather the second, third, or fourth highest peak corresponds to the correct solution. An example of this situation is shown for node #45 of the synchronized dataset (second plot in Figure 11). Node #45 is very near to the abutment and therefore experiences the least vibrations, thereby resulting in a very low signal to noise ratio. Note that the correct solution corresponds to the fourth (and last) peak. Such nodes can be identified either by examining the resulting mode shapes or by the presence of multiple peaks in the KDF. In such a case, iterating through all peaks and observing the mode shape can help identify the correct time delay. Such situations can be avoided by using a ‘clean’ portion of the measurement that is free of high amplitude vibrations and periodic noise.

### 3.3. Results of the Proposed Algorithm

The synchronization algorithm is applied on the de-synchronized dataset with the first ten modes used for superposition. Most of the nodes have a single-peak KDF and a few nodes have a multiple-peak KDF, in which case the highest peak was selected as the first choice for solutions. Figure 11 shows the frequencies of the first ten modes and the first two mode shapes after the first iteration, that is, after selecting only the top ranked solutions for all nodes.

**Figure 12:**
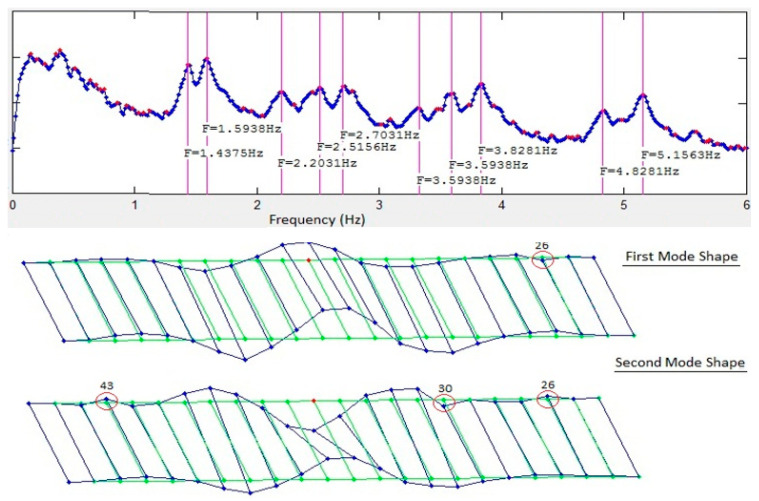
First solution set after the first iteration of the algorithm.

**Figure 13:**
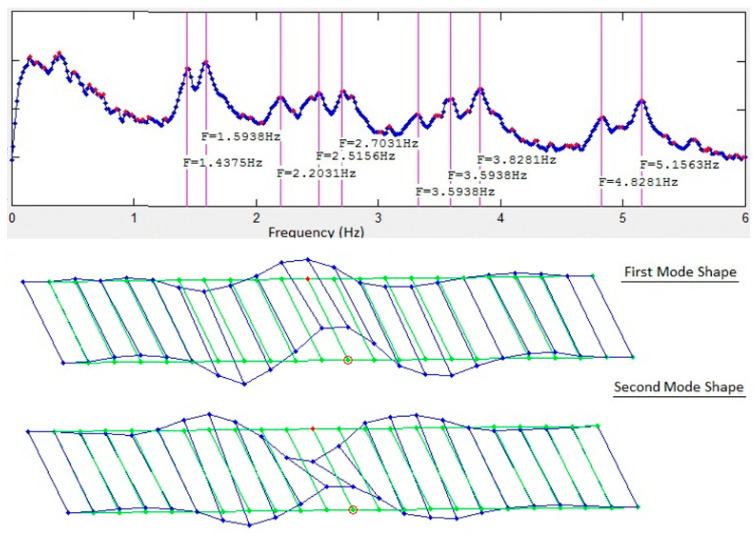
Final solution set obtained by selecting the second solutions for nodes #26, #30, and #43.

Comparison of the mode shapes in Figure 12 for the first solution set with those in Figure 3 and Figure 4 shows that a significant improvement has been made in the first iteration. Nodes #26, #30, and #43 can be readily identified as being in the wrong position (Figure 12). These three nodes have multiple peaks in their KDFs and selecting the second peak of the KDFs for these nodes gives the second solution set. The resulting mode shapes from the second solution set are shown in Figure 13. Comparing the second solution set in Figure 13 with Figure 3 shows that the synchronization algorithm has successfully estimated the time delays introduced in the de-synchronized dataset and two iterations of the proposed algorithm and has resulted in the estimation of correct mode shapes with the de-synchronized data.

## 4. Application of the Algorithm to a 30-Story RC Building

The original motivation for this study was to synchronize the data from AVTs of large buildings. These are buildings for which it is not practical (or even feasible) to collect data from all nodes of the building simultaneously. The proposed synchronization algorithm assumes that, in every setup, there exists a lag between the reference sensor and each of the roving sensors and that this time lag is small, i.e., under 2 s.

### 4.1. Data Acquisition and Structural Identification

**Figure 14:**
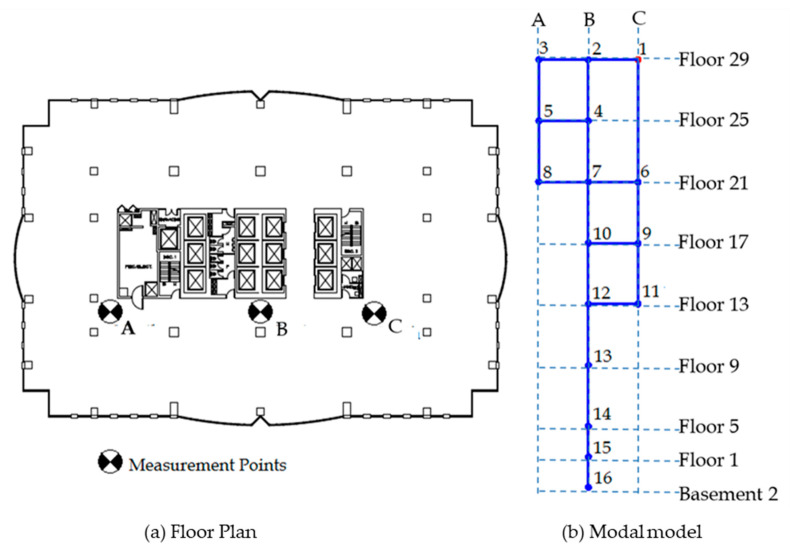
(**a**) Typical floor plan of the test building showing location of sensors and (**b**) modal model that resulted from the choice of measurement points.

The test building is a 30-story, reinforced concrete (RC) shear wall building with a rectangular plan with dimensions 37 m by 55 m. The building’s height above the ground is approximately 122 m. The building was located in downtown Montreal, QC, Canada and constructed in 1988. The main lateral load resisting system consists of an RC shear wall in the middle of the building, housing elevator shafts and ventilation ducts. Figure 14a shows a typical floor plan of the building and locations of sensor placement.

For the AV test in the building, translational velocities resulting from ambient excitations were measured using two Lennartz LE3D/5s triaxial seismometers, each connected to a separate LEAS CitySharkII DAS. These sensors measure velocities in three orthogonal directions: two in the horizontal plane and one in the vertical direction. However, only the two horizontal velocities were considered for further analysis since the vertical motion was not of interest for this study.

Due to access restrictions, only part of the corridor around the central elevator shafts was available for the test. Three points were chosen for placement of sensors on floors 13 and above, designated as points A, B, and C, as shown in Figure 14a. On all the other floors, only one point (B) was available for sensor placement. The reference sensor was placed on the 29^th^ floor at point C, and every fourth floor was measured down to the ground floor (floor 1) and the second basement. The data from three points (Point C on 25^th^ floor and point A on 13^th^ and 17^th^ floors) were later found to have been corrupted (unable to read data from the DAS) and were thus removed from the analysis. This resulted in a 16-node modal model, as shown in Figure 14b. The dotted blue line shows the grid, while the solid blue lines and blue dots show the modal model. The red dot indicates the position of the reference node/sensor.

Both DAS were connected to their GPS receivers immediately before the commencement of the test and their internal clocks, which are accurate to 1 millisecond, were synchronized to the GPS satellites. For the first five setups, the DAS could be synchronized by a remote-control starter (RCS), a device that sends a trigger signal to both DAS over radio frequency to enable synchronous recording. However, the range and attenuation of radio signals through concrete slabs did not allow the use of RCS for other setups. The records in these setups were tentatively synchronized with the GPS time markers. For all setups, five minute records were taken at the sampling frequency of 1000 Hz. Such a high sampling frequency is not required for AVT of buildings, but to use GPS time markers for synchronization, the data has to be sampled at the same sampling period as the accuracy required, i.e., 1 millisecond. For the purposes of system identification analysis, the data was resampled at a decimated sampling frequency of 100 Hz. This procedure also reduces the energy of noise in the power spectrum of the data.

**Figure 15:**
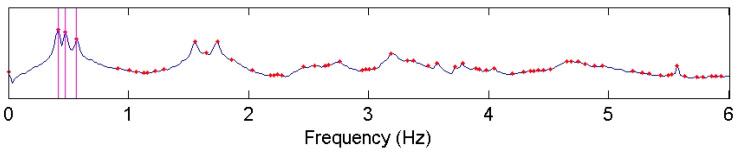
Plot of the first singular values for test building. The first three frequencies are marked by vertical lines.

FDD was used for analyzing the AVT data. Figure 15 shows the plot of the first singular values (SV) obtained by the FDD method. The first three frequencies can be readily identified as clear peaks as shown by vertical lines.

**Figure 16:**
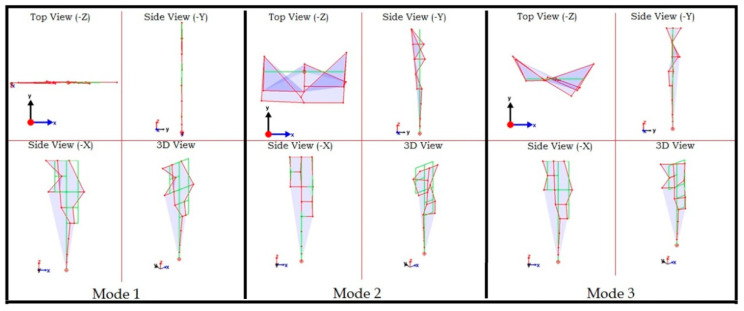
First three mode shapes: unsynchronized dataset.

The mode shapes corresponding to the three frequencies are shown in Figure 16. Each mode is shown in three orthogonal and one isometric view. The green lines show the undeformed shape, while the red lines show the undeformed shape. It can be observed that data on almost all nodes is unsynchronized and result in incorrect mode shapes. Next, the proposed synchronization method is applied to the three mode shapes to synchronize the data and obtain correct mode shapes.

### 4.2. Application of the Proposed Time Synchronization Algorithm

**Figure 17:**
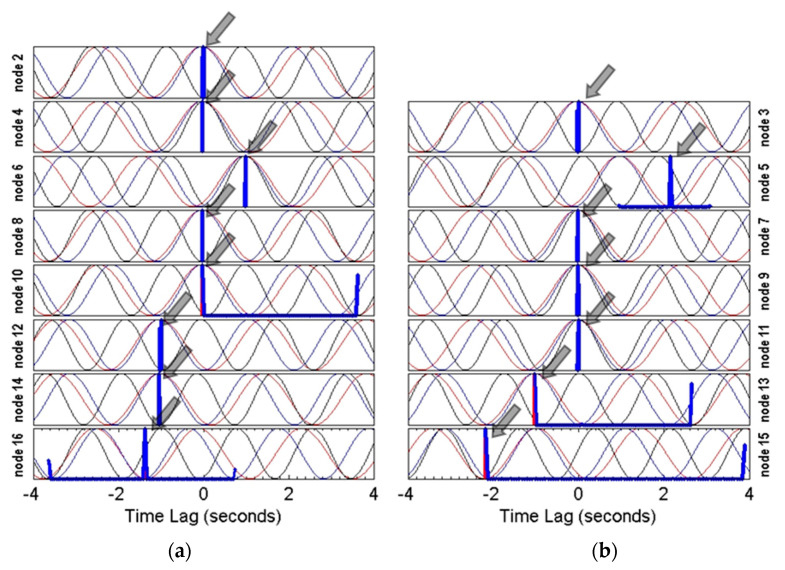
PATL (**a**) and KDF (**b**) plots for all the nodes in the building. Note that most of the nodes have single-peaked KDF. Arrows show the top ranked solutions selected for the first iteration.

It is observed from the PATL plots for roving nodes in the model along with KDF plots (in blue lines) in Figure 17 that, since, nodes #2–4 were synchronized using RCS, there is only one peak in the KDF correctly indicating the solution corresponding to zero-time lag. Additionally, for most of the nodes, the KDF plots suggest only one solution. For nodes #7, #8, #9, and #11, there is no time lag.

**Figure 18:**
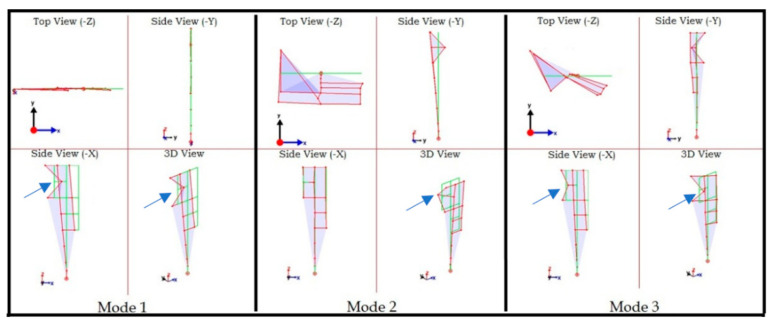
First three mode shapes after applying the time lags of the first iteration. (Note that node #5, identified by an arrow in the side view diagrams, seems to be out of synchronicity).

**Figure 19:**
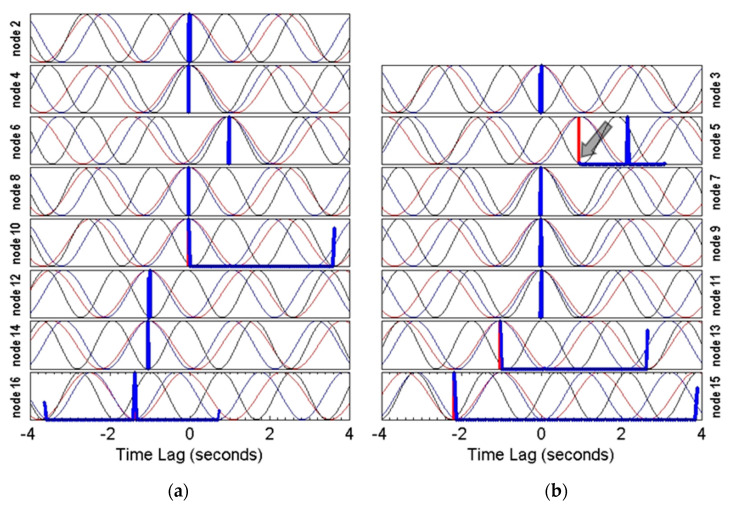
PATL (**a**) and KDF (**b**) plots for all nodes in the building. The arrow shows the second ranked solutions for node #5.

As a first iteration, the top ranked solutions suggested by the KDF plots were selected for all nodes. After applying the time lags from the selected solutions, the cross power spectral density matrix was re-calculated and mode shapes were extracted from singular value decomposition [11]. Figure 18 shows the three mode shapes after the first iteration. Except for node #5, all the nodes seem to be in synchronicity suggesting that the top ranked solutions generated the correct time lags. In order to synchronize node #5 in the second iteration, the second ranked solution was selected from the KDF plot as shown in Figure 19.

**Figure 20:**
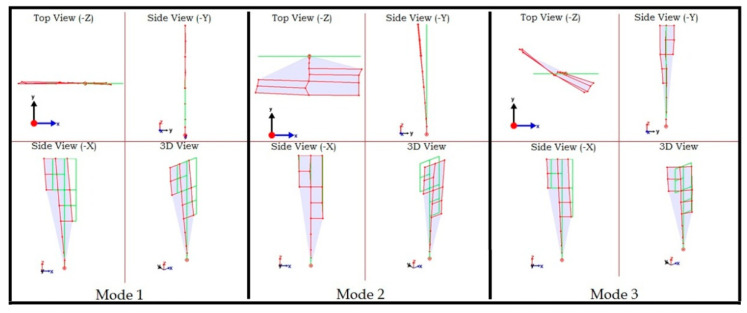
The first three mode shapes after the second iteration. All nodes are synchronized.

After the application of second iteration the data was re-processed to extract the first three mode shapes shown in Figure 20. All nodes are now synchronized, and the mode shapes can be correctly interpreted as the first fundamental modes in Y, X, and torsional directions.

**Figure 21:**
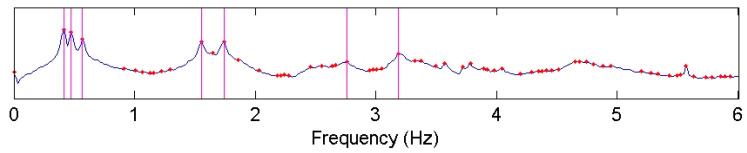
Plot of first singular values showing seven identified frequencies.

**Figure 22:**
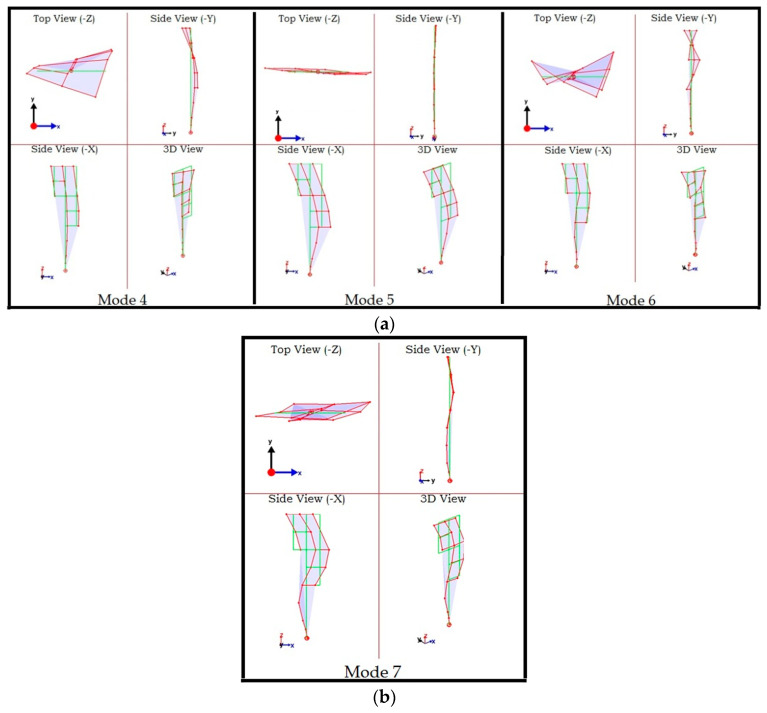
Additional modes after synchronization process: modes 4, 5, and 6 (**a**) and mode 7 (**b**).

Once the data has been synchronized, additional modes can be obtained from the data. Figure 21 shows the SV plot of the synchronized data (this plot is like that of the unsynchronized data) with four additional modal frequencies marked. Figure 22 shows the mode shapes corresponding to the four additional modal frequencies. Modes 4 and 6 are the second and third torsional modes, respectively. Modes 5 and 7 are second and third flexural modes in the X-direction. Additionally, the fundamental mode is the first flexural mode in the X-direction while the second mode is the first flexural mode in Y-direction. This suggests that the building is stiffer in Y-direction compared to the X-direction contrary to what may be expected observing the planar geometry of the building, which implies that the flexural modes in Y-direction should have been attained at relatively lower frequencies. This is because, comparatively greater stiffness is contributed in the Y-direction by the shear walls, which is a main structural component of the building, as shown in Figure 14a.

Although RCS and GPS time markers were used for synchronization of the data collected from the AVT of the building, it was observed that the GPS time markers are not always accurate, and that the data thus obtained can be unsynchronized. This creates a potential problem in the implementation of AVTs in large buildings such as medium- and high-rise buildings or buildings with very large plans. Using long cables to connect all sensors to the same DAS is not only expensive but also deteriorates the signal-to-noise ratio in the data. In extreme cases, it may simply not be possible to connect sensors from far away locations to a single DAS.

## 5. Conclusions

The use of multiple DAS is necessary in the output-only modal analysis of medium- and high-rise buildings to avoid complicated meshes of cables arising from using a single DAS for simultaneous ambient vibration measurement which can potentially disrupt the operations of the building. Nevertheless, it introduces the time synchronization discrepancies in the vibration measurement which can result in erroneous or inconclusive results of output-only modal analysis. In this research, first the issue of time synchronization was artificially introduced in synchronous data obtained during a bridge testing as such data is extremely difficult to obtain for a high-rise building. It was observed that time synchronization deficiencies influence mode shapes or phase angles but not the eigen values. This information was exploited and back calculations were performed to obtain the exact time lag which introduce these deficiencies in the phase angles or mode shapes. These time lags were then compensated to rectify the issues in the mode shapes associated with the phase angles. Next, the AVT is performed on a 30-story building using multiple DAS, and hence the time synchronization discrepancies were expected to be present in the datasets thus obtained. The phase angle compensation explained above is performed to rectify the mode shapes obtained for the building. To increase the robustness of the proposed algorithm to the inherent inconsistencies in these datasets, the kernel density function is applied to rank multiple time shift estimates that are sometimes detected by the algorithm when inaccuracies exist in the data arising from low signal-to-noise ratio and/or presence of colored noise in the ambient excitations. The proposed algorithm solves a practical problem of time synchronization discrepancies in high-rise buildings in the output-only modal analysis of medium- and high-rise buildings.

## Data Availability

The data can be made available upon research request and subject to permission from the owners of the structures at which the measurements were performed.
